# Quality of life in women with normal pregnancy

**DOI:** 10.1038/s41598-024-63355-7

**Published:** 2024-05-30

**Authors:** Małgorzata Wójcik, Bartosz Aniśko, Idzi Siatkowski

**Affiliations:** 1 Department of Physiotherapy, Poznan University of Physical Education, Faculty of Sport Sciences in Gorzow Wlkp., 61-871 Poznan, Poland; 2 Department of Mathematical and Statistical Methods, Poznan University of Life Science, 60-637 Poznan, Poland

**Keywords:** Quality of life, Rehabilitation, Health care

## Abstract

Pregnancy affects a woman’s physiological and psychological state. One of the most important aspects that requires attention is the quality of life of pregnant women. The quality of life of women during this period is influenced by a number of factors, such as back and pelvic pain, physiotherapy and physical activity, and also sexual satisfaction. Eighty-five women aged 21–40 years (30.80 ± 5.05) in pregnancy trimesters participated in the study: 17 women were in the first trimester, 32 women were in the second, and 36 women in the third trimester. The World Health Organisation Quality of Life (WHOQOL-BREF), Oswestry Disability Index (ODI) and the Sexual Satisfaction Scale for Women SSS-W-R15 were used to answer the research hypotheses. Respondents also provided information on questions regarding physiotherapy treatments and physical activity. Quality of life (WHOQOL-BREF) and disability due to back pain (ODI) showed a statistical association and relationship (p-value = 0.045, rho = − 0.22). Quality of life (WHOQOL-BREF) has an association with sexual satisfaction in pregnant women (SSS-W-R15) (p-value = 0.003, rho = 0.32). The trimester of pregnancy has an effect on ODI (p-value = 0.027). A significant effect occurred in a detailed comparison between the first and third trimesters of pregnancy (p-value = 0.026). The trimester also has an impact on quality of life (WHOQOL-BREF) (p-value = 0.002). In a detailed analysis, a significant effect occurred between the first and third trimesters of pregnancy (p-value = 0.001). Moreover, the trimester of pregnancy has an impact on sexual satisfaction (SSS-W-R15) (p-value = 0.027). After detailed statistical analysis, a significant effect occurred between trimesters one and three of pregnancy (p-value = 0.046). On the other hand, the number of days of physical activity performed by pregnant women per month and the type of physical activity had no effect on the ODI (p-value = 0.071). The type of physical activity performed by pregnant women also has no effect on ODI (p-value = 0.023). The number of physiotherapy treatments used has no effect on the ODI (p-value = 0.156). Type of physiotherapy treatment has no effect on ODI (p-value = 0.620). Normal pregnancy quality of life (WHOQOL) is related to ODI and sexual satisfaction (SSS-W-R15), while the trimester of pregnancy also has an impact on quality of life, disability due to back pain and sexual satisfaction.

## Introduction

The impact of pregnancy on women's quality of life is an important area of research in the field of medicine, including physiotherapy. Hormonal changes, physical discomfort and issues related to sexual life are just some of the aspects that affect women's lives during pregnancy. A better quality of life for pregnant women is influenced by factors such as the mother's average age, primiparity, early gestational age, absence of economic problems, high level of education, employment, marriage, having family and friends^1^. One of the important factors negatively affecting the quality of life of pregnant women is spinal pain. Studies have shown that pregnant women are more likely to experience lumbar back pain than women who have never been pregnant^2,3^. Back pain in women before pregnancy occurs sporadically, whereas during pregnancy a significantly higher incidence of back pain was observed^2^. Lower back pain usually starts as early as the first or second trimester of pregnancy^2,4,5^. Pregnancy has also been shown to affect posture, leading to an increase in lumbar lordosis and thoracic kyphosis^3,5^ and a deterioration in gait quality and balance ability, which is associated with changes in the centre of gravity and postural changes^3^. Back pain can affect sleep disturbance, which in turn will affect quality of life^6–9^.

Pregnant women's sex lives also change. Half of women experience a decline in sexual interest during pregnancy^10,11^. Most often, the greatest decline or complete cessation of sexual activity occurs during the third trimester of pregnancy^12^.

Physical activity may be a way to improve the quality of life of pregnant women. Up to 55% of women discontinue physical activity during pregnancy, while 30% choose to continue physical activity and 15% choose to reduce its intensity^13^. Physical activity during pregnancy can help reduce the risk of becoming overweight or obese after childbirth^14,15^ and the onset of gestational diabetes^16–18^. Moreover, engaging in physical activity can help reduce back pain^19^ reduce the risk of urinary incontinence^20,21^ or lower the risk of anxiety and depression^22^.

As pregnancy is a dynamic period in a woman's life due to the developing baby and the changes taking place in the woman's body. The aim of the study was to assess the quality of life of healthy pregnant women over a 4-week period. Before proceeding with the study, the following research hypotheses were established:

1. The quality of life of women with normal pregnancy is related to disability caused by lumbosacral spine pain complaints and sexual satisfaction.
2. The trimester of pregnancy affects the incidence of disability caused by lumbosacral spine pain complaints and quality of life and sexual satisfaction.
3. The number of days of physical activity performed by pregnant women on a monthly basis and the type of physical activity (walking, exercise and swimming) has an impact on the incidence of disability caused by lumbosacral spine pain complaints.
4. The number of physiotherapy treatments (therapeutic massage, exercise, manual therapy and stretching) used on a monthly basis has an impact on the incidence of disability caused by lumbopelvic spine pain complaints.
5. The type of physiotherapy treatments (therapeutic massage, exercise, manual therapy and stretching) used has an influence on disability caused by lumbosacral spine pain complaints.

## Materials and methods

### Design

The study was conducted within the framework of the project “Quality of life and functional relationships of the temporomandibular and pelvic joints in women with normal pregnancy”. The quality of life of women in the previous 4 weeks of their life was taken into account.

### Sample

The study participants were 85 women with normal pregnancy, aged 21–40 years (30.80 ± 5.05), weight 73.43 ± 5.08, height 166.68 ± 6.33. Pregnancy trimesters were taken for statistical analysis. There were 17 women in the 1st trimester of pregnancy (BMI 22.32 ± 2.85), 32 women in the 2nd trimester of pregnancy (BMI 28.16 ± 4.69) and 36 women in the 3rd trimester (BMI 24.77 ± 7.25).

### Procedures

The study used standardized questionnaires, including the World Health Organization Quality of Life (WHOQOL-BREF), Oswestry Disability Index (ODI) and the Sexual Satisfaction Scale for Women SSS-W-R15 in the Polish language version. Study participants were asked to mark their answers on the questionnaires and scales anonymously.

The WHOQOL-Bref questionnaire consists of 26 questions. These concern self-assessment of the overall quality of life and general health of pregnant women. The remaining 24 questions assess four domains: physical health—7 questions; psychological—6 questions, social relations—3 questions and environmental—8 questions. The questionnaire has a five-point rating scale from 1 to 5 points: the higher the number of points, the better the quality of life, which in each domain was expressed as average values, calculated according to the key and guidelines^23^. The Polish version of the questionnaire was used in the study. For the purposes of this publication, we considered the overall score.

The Oswestry Disability Index (ODI) questionnaire was developed to assess how thoracolumbar pain affects people's ability to cope with their daily life. The ODI contains 10 items relating to: pain intensity, personal care, lifting, walking, sitting, standing, sleeping, social life, traveling and homemaking. For each item, the taker gives an answer to six statements with a score from 0 to 5, with 0 representing the least disability and 5 representing the most disability. Scores for all questions answered were summed (range 0–50). Zero means no disability, and 50 means maximum disability. Scores are interpreted as: no disability (0–4 points); minor disability (5–14 points); moderate disability (15–24 points); severe disability (25–34 points) and total disability (35–50 points)^24^. The Polish version of the ODI24 questionnaire was used. For the purposes of this work, we considered the overall score without analyzing individual domains.

The Sexual Satisfaction Scale for Women SSS-W-R15 consists of 15 questions dealing with satisfaction, communication and adjustment. Each question is scored from 1 to 5, where individual scores mean: 1—strongly disagree; 2—rather disagree; 3—hard to say; 4—rather agree and 5—strongly agree. The sum of the three scales obtained (satisfaction—score 5–25, communication—score 4–20 and matching—score 6–30) makes up the global sexual satisfaction score^25^. The higher the point value, the better the respondent’s intimate life. The scores were calculated according to the key. The Polish version of this scale was used. For the purposes of this study, we took into account the total score without analyzing the individual scales.

The pregnant women surveyed were asked questions such as: what physiotherapy treatment was performed for thoracolumbar pain, and how many treatments were performed over the past month (4 weeks), as well as whether the women were physically active (how many days over the past month) and what type of activity they performed. The study participants were recruited using flyers and posters. All participants were informed about the study protocol by means of an information sheet and their written consent to participate in the study was obtained. The women were screened by interview to verify their eligibility according to the inclusion and exclusion criteria. Inclusion criterion: woman with a normal pregnancy and voluntary participation in research. Exclusion criterion: pathological pregnancy, history of back pain before pregnancy, postural defects, scoliosis, injuries, illnesses and medication use, and lack of consent in the study. 150 pregnant women applied to participate in the study; however, due to the inclusion and exclusion criterion, 85 women were finally qualified to participate in the study. Data collection took 8 weeks.

Participants completed standardized questionnaires and answered the questions once and did so anonymously. The physiotherapy treatments used were therapeutic massage, exercise, manual therapy and stretching. The physical activities undertaken by the pregnant women were walking, exercise and swimming.

### Statistics

As normal distribution was not met for the data obtained, non-parametric tests were used. Spearman's rank correlation test was used to check whether quality of life is related to disability due to lumbosacral spine pain and the sexual satisfaction scale.

We used the Kruskal–Wallis test and the POST-HOC Dunn test with Bonferroni correction to test whether the trimester of pregnancy influences the incidence of disability due to lumbosacral spine pain and the quality of life and sexual satisfaction of the pregnant women.

Again, the Kruskal–Wallis test and Dunn test were used to test whether the number of days per month of physical activity performed by the women affects the incidence of disability because of lumbosacral spine pain, and the exact Fischer test for contingency tables to test whether the type of physical activity performed by the pregnant women affects the incidence of disability due to lumbosacral spine pain.

The Kruskal–Wallis test and Dunn test were also used to test whether the number of physiotherapy treatments used per month and the type of physiotherapy treatments used affect disability caused by lumbosacral spine pain. All calculations, statistical analyses and figures were performed using the R software version 4.3.2^26^.

### Ethical approval and consent to participate

All procedures performed in studies involving human participants followed the ethical standards of the institutional and/or national research committee along with the 1964 Helsinki declaration and its later amendments or comparable ethical standards. The study protocol was approved by the Bioethics Committee of Poznan University of Medical Sciences (permit No. KB/982/23).

## Results

First, statistical analysis was performed to see if quality of life expressed by the WHOQOL-BREF questionnaire is related to ODI. The result of Spearman's rank correlation coefficient rho = − 0.22 indicates a weak negative relationship. Moreover, the correlation between WHOQOL-BREF and ODI is statistically significant (p-value = 0.045).

The Sexual Satisfaction in Women Scale SSS-W-R15 was used to check whether quality of life is related to sexual satisfaction in pregnant women. The strength of the relationship is weak, and the direction of the relationship is positive (rho = 0.32). In addition, the relationship between SSS-W-R15 and ODI is significant because p-value = 0.003.

**Figure 1:**
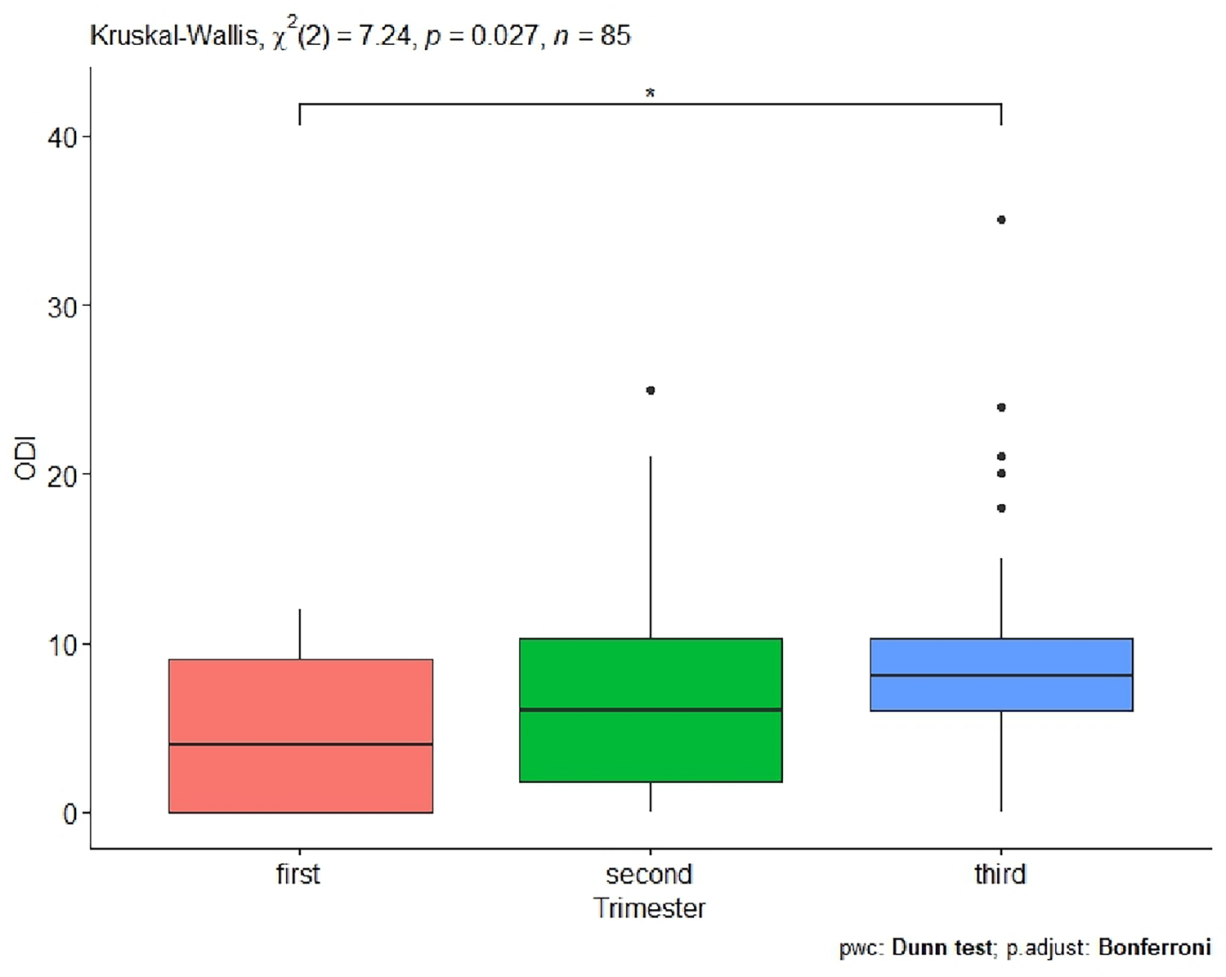
Trimesters of pregnancy and disability due to lumbosacral spine pain expressed by the Oswestry Disability Index questionnaire (significant codes *p-value < 0.05).

The next step in the statistical analysis was to examine in detail whether the trimester of pregnancy has an effect on ODI. The resulting p-value = 0.027 indicates that the trimester of pregnancy has an effect on ODI. In addition, a detailed comparison was made between trimesters of pregnancy in women (Table 1, Fig. 1). In the detailed statistical analysis, a significant effect occurred when comparing the first and third trimester of pregnancy p-value = 0.026 (Table 1, Fig. 1).

**Table 1:** Comparison between the trimesters of pregnancy in women and their effect on disability caused by lumbosacral spine pain expressed by the Oswestry Disability Index (ODI) questionnaire; comparison between the trimesters of pregnancy in women and the impact on quality of life as expressed by the WHOQOL-BREF questionnaire; comparison between trimesters of pregnancy in women and the effect on sexual satisfaction expressed by the SSS-W-R15 questionnaire (significant codes *p-value < 0.05, significant codes, **p-value < 0.01).

Questionnaire	Trimester of pregnancy First–second p-value	Trimester of pregnancy First–third p-value	Trimester of pregnancy Second–third p-value
ODI	0.570	0.026*	0.355
WHOQOL-BREF	0.052	0.001**	0.590
SSS-W-R15	0.450	0.046*	0.045

**Figure 2:**
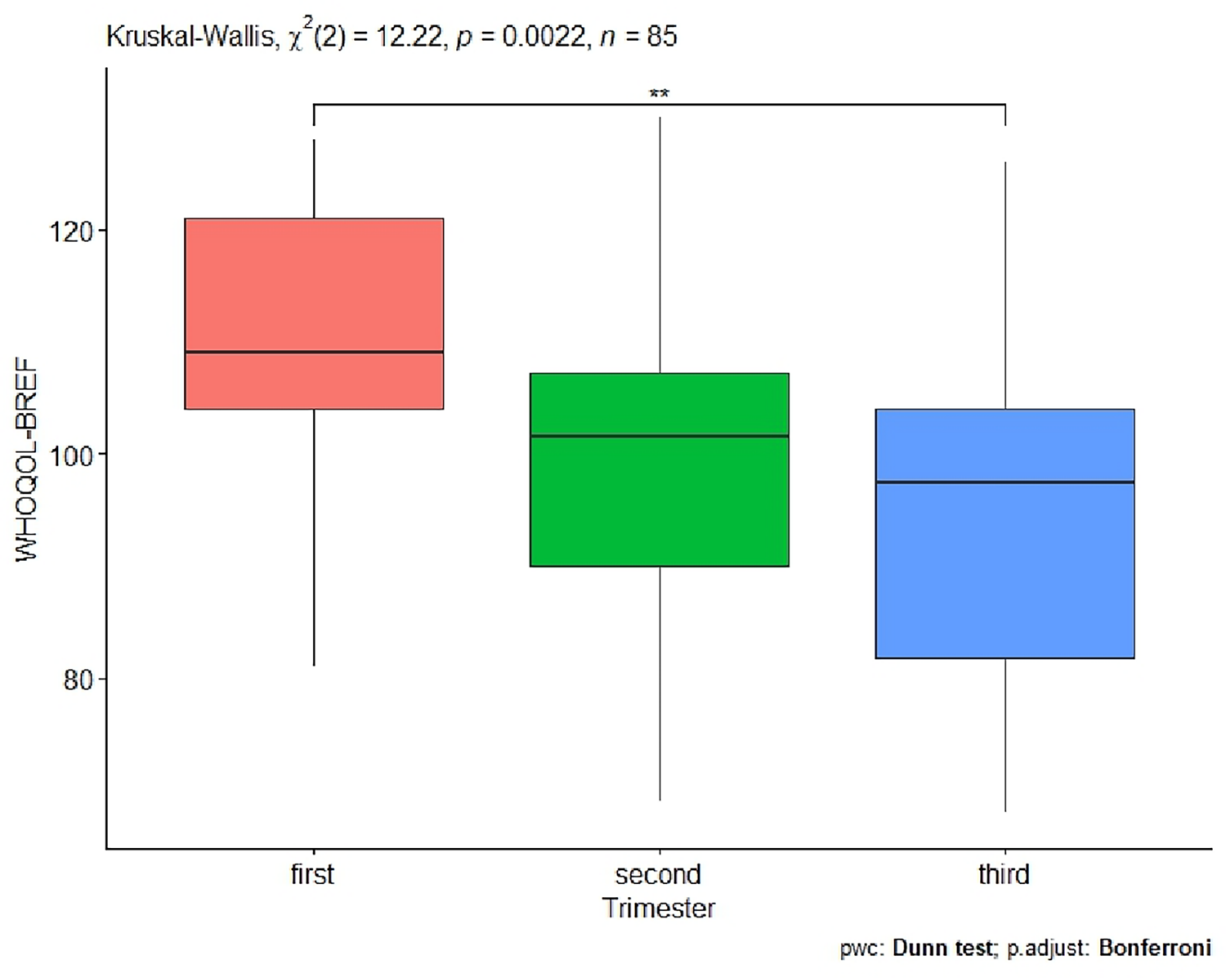
Women’s trimesters of pregnancy and quality of life as expressed by the WHOQOL-BREF questionnaire (significant codes **p-value < 0.01).

Next, the issue of the effect of the trimester of pregnancy on quality of life was investigated. The resulting p-value = 0.002 indicates that the trimester of a woman's pregnancy has an impact on their quality of life. Next, a detailed analysis was conducted between the trimesters of pregnancy in women (Table 1, Fig. 2). In the detailed statistical analysis, a significant effect occurred when comparing the first and third trimester of pregnancy p-value = 0.001 (Table 1, Fig. 2).

**Figure 3:**
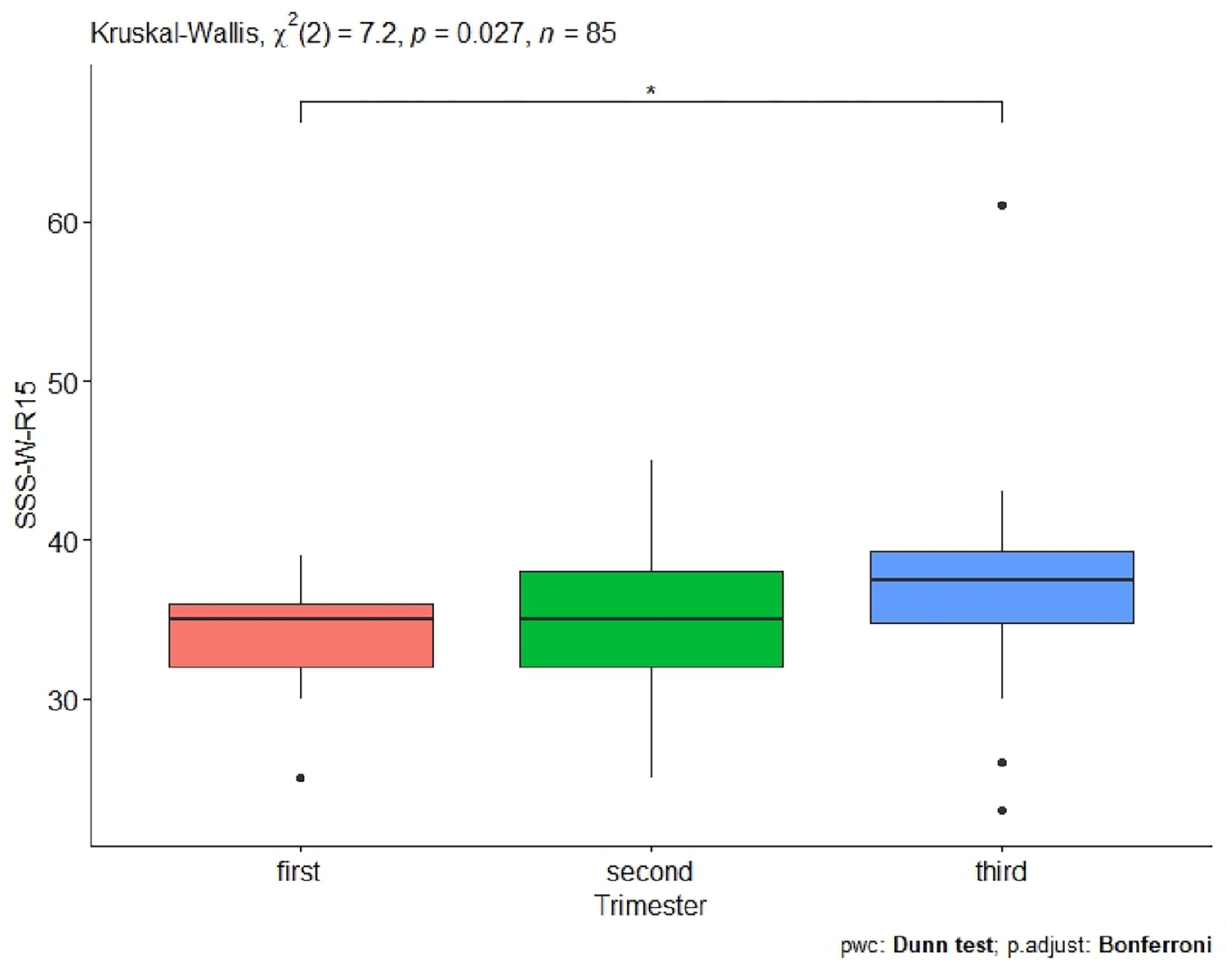
Women’s trimesters of pregnancy and sexual satisfaction as expressed by the SSS-W-R15 questionnaire (significant codes *p-value < 0.05).

Further statistical analysis examined whether the trimester of pregnancy has an effect on sexual satisfaction as expressed by the women in the SSS-W-R15 questionnaire. The value obtained indicates that the trimester of pregnancy has an effect on sexual satisfaction because p-value = 0.027. After detailed statistical analysis, it is clear that a significant effect occurred when comparing the first and third trimester of pregnancy p-value = 0.046 (Table 1, Fig. 3).

Next, we investigated the issue of whether the number of days of physical activity performed by pregnant women per month and the type of physical activity has an effect on the incidence of disability due to complaints of lumbosacral spine pain. The resulting p-value = 0.071 indicates that the number of days of physical activity performed per month has no effect on the incidence of disability caused by lumbosacral spine pain complaints.

Moreover, the type of physical activity performed by pregnant women has no effect (p-value = 0.023) on the incidence of disability due to complaints of lumbosacral spine pain.

A further statistical analysis examined whether the number of physiotherapy treatments used has an effect on disability due to lumbosacral spine pain. The result indicates non-significance p-value = 0.156.

The final step in the statistical analysis was to examine the issue of whether the type of treatment, i.e., massage, exercise, manual therapy or stretching, has an effect on disability caused by lumbosacral spine pain. The resulting p-value = 0.620 indicates a lack of statistical significance.

## Discussion

These results showed that the quality of life of pregnant women is related to disability due to thoracolumbar back pain and satisfaction with sexual life. The definition of quality of life was formulated by the World Health Organisation’s (WHO) Quality of Life Group (WHOQOL Group), which defined quality as an individual's perception of his or her position in life in the context of the culture and value systems accepted by the society in which he or she lives and in relation to his or her life goals, expectations and interests^27^.

Interpreting the quality of life of pregnant women can be perplexing, as various questionnaires are used to assess it, such as QOL (HRQoL)^28,29^, 36-Item Short-Form Health Survey (SF-36)^30^ and the World Health Organization Quality of Life questionnaire (WHOQOL-Bref)^23^. However, assessing the quality of life of pregnant women can play a significant role with regard to prenatal care. Positive factors influencing pregnant women's quality of life include maternal age, primiparity, early gestational age, absence of social and economic problems, having family and friends, exercising, a feeling of happiness during pregnancy and optimism^31^. Factors causing poorer quality of life include medically assisted reproduction, complications before or during pregnancy, obesity, nausea and vomiting, epigastric pain, back pain, smoking in the months before conception, alcohol dependence, sleep difficulties, stress, anxiety, depression during pregnancy, sexual or domestic violence^31^.

Sexual activity is not just for reproduction purposes but is a form of communication between partners. Women who have sex during pregnancy are likely to have higher self-esteem, adjust more quickly to pregnancy and enjoy emotional intimacy and marital cohesion more^32–34^. Sexual problems that arise during pregnancy can disrupt marital harmony, thus negatively affecting the quality of sexual life, as well as family and social life^35,36^. The occurrence of musculoskeletal pain, including pain in the spine, has a negative impact on women's sexual activity^37–39^. The results obtained in this study also indicated that the trimester of pregnancy has an impact on the incidence of disability caused by lumbosacral back pain and also on both quality of life and sexual satisfaction. Back pain can be caused by pregnancy, ovarian cysts, uterine prognathism, endometriosis, uterine myomas or inflammation of the upper genital tract^40^. Increased abdominal tension can shorten the iliac-lumbar muscle and consequently cause lower back dysfunction^40^. During pregnancy, the developing baby in utero rests on the lumbar muscles, thus affecting their burden^41^. Furthermore, the abdominal muscles, including the rectus abdominis, are stretched^42^. During pregnancy there is a muscular imbalance in the muscles of the trunk and especially the abdomen^42^. Pain in the pelvic area that radiates to the lumbar spine can also cause inflammation of the female reproductive organs; this condition in pregnant women can be treated non-pharmacologically by means of balneoclimatology treatments^43^. Visceral manipulation combined with a personal physiotherapy program can be an effective way to reduce pain sensations and improve mobility of the lumbar spine^44^. Visceral therapy is also effective in treating non-specific back pain^45–47^.

The statistical analysis in our study shows that the number of days of physical activity performed by pregnant women per month and the type of physical activity do not affect the incidence of disability caused by lumbosacral spine pain. However, the literature reports that physical activity is a good means of improving physical and mental health^48^. Positive effects are also observed in pregnant women^49^, but despite this, pregnancy is one of the reasons for limiting physical activity among women^50^. In its 2020 guidelines, the WHO recommends that women who were physically active before pregnancy should continue with this during pregnancy and the postpartum period, as long as there are no health contraindications^51^. Physical activity during pregnancy and the postpartum period is beneficial to the health of both mother and child because it reduces the risk of pre-eclampsia, gestational hypertension, gestational diabetes, excessive weight gain during pregnancy, postpartum complications and postpartum depression, as well as minimizing the risk of stillbirth and complications in the newborn^51,52^.

The WHO recommends that adults undertake 150–300 min of moderate-intensity physical activity or 75–150 min of high-intensity physical activity or an equivalent combination of moderate- and high-intensity aerobic physical activity each week^51^. On the other hand, pregnant and postpartum women with no contraindications to physical activity are advised to do the following: regular physical activity throughout pregnancy and the postpartum period, including at least 150 min of moderate-intensity aerobic physical activity throughout the week to achieve significant health benefits, and to include aerobic and muscle-strengthening exercises and stretching exercises^51^.

Pregnant women are advised to be physically active to minimize the effects of a sedentary lifestyle, including the occurrence of overweight or obesity and the health consequences of these conditions^51,52^. Zhang et al. noted that doing physical activity during pregnancy has an impact on the path of labor, reducing the number of Cesarean sections performed^53^. Among the recommended forms of exercise for pregnant women are walking, jogging, stretching exercises, yoga, balance exercises, swimming, cycling, and aerobics for pregnant women^54,55^. Some researchers also found an increase in the performance of activities during pregnancy by women in the first and second trimesters^56,57^. In contrast, other researchers noted a decline in physical activity undertaken by women in the third^58^ and observed a decreasing percentage of physically active women in subsequent trimesters of pregnancy^59^. Borodulin et al. found that the overall level of physical activity decreased non-significantly between the 17–22 and 27–30 weeks of pregnancy and 27–30^60^.

Our results also showed that the number of physiotherapy treatments used per month has no effect on disability caused by lumbosacral spine pain complaints. Moreover, the type of physiotherapy treatments used (therapeutic massage, manual therapy, exercise, stretching) has no effect on disability caused by lumbosacral spine pain complaints. In 2023, Kandru et al. published a literature review that included 16 studies evaluating the effectiveness of exercise in treating lower back and pelvic pain in pregnant women^61^. Their results testified to the effectiveness of exercises such as pelvic stabilization exercises, yoga, Pilates or stretching exercises in managing pain in pregnant women^61^. A systematic review performed by Liddle in 2015 evaluated selected methods used in physiotherapy for effectiveness in preventing and treating pelvic and lower back pain^62^. Methods demonstrating efficacy included exercise and osteopathic manual therapy^62^. A study conducted by Mamipour aimed to evaluate the effects of core stability exercises on pain, functional disability and quality of life in pregnant women with lumbar and pelvic pain. The study comprised 35 pregnant women with lumbar and pelvic pain, who were randomly assigned to either a study group or a control group^63^. Stabilization exercises were performed in 10 series of 10 s each. The results showed significant improvements in pain, functional disability and quality of life in the exercise group compared to the control group^63^. According to the recommendation made by Esther van Benten et al., in the treatment of spinal pain in pregnant women, physiotherapists should recommend more physical activity and strength training, abdominal muscle strengthening exercises, pelvic and gluteal muscle strengthening exercises^64^.

In addition, the recommendations also include the use of manual therapy and therapeutic massage. The authors pay special attention to the need to educate the patient about physiotherapy and exercise, as well as adopting an individual approach to each patient^64^. It is worth noting that women have a low awareness of physiotherapy and of the use of exercise during pregnancy^65,66^.

## Conclusions

The study confirmed that women’s quality of life is related to disability caused by lumbosacral spine pain and sexual satisfaction, and the trimester of pregnancy has an impact both on the occurrence of disability and on quality of life and sexual satisfaction.

The number of days of physical activity performed by pregnant women per month has no impact on the incidence of disability due to lumbosacral spine pain, nor does the type of physical activity have any effect.

On the other hand, the number of physiotherapy treatments used per month and the type of physiotherapy treatments used have no impact on disability caused by lumbosacral pain.

### Limitations of the study

One limitation of the study is the small sample of women surveyed. For the purpose of this publication, the total score from the questionnaires and the scale were taken into account without considering the individual domains, which will be taken into consideration in future publications. Another limitation may be that the observations were conducted only for 4 weeks of the women's pregnancies.

## Data Availability

The datasets used and analysed during the current study available from the corresponding author on reasonable request.
